# A novel 1-bp deletion variant in *DAG1* in Japanese familial asymptomatic hyper-CK-emia

**DOI:** 10.1038/s41439-022-00182-0

**Published:** 2022-01-27

**Authors:** Luoming Fan, Shiroh Miura, Tomofumi Shimojo, Hirotoshi Sugino, Ryuta Fujioka, Hiroki Shibata

**Affiliations:** 1 Division of Genomics, Medical Institute of Bioregulation, Kyushu University, Fukuoka, Japan; 2 Department of Neurology and Geriatric Medicine, Ehime University Graduate School of Medicine, Ehime, Japan; 3 Sugino Pediatric Clinic, Hiroshima, Japan; 4 Department of Food and Nutrition, Beppu University Junior College, Oita, Japan

**Keywords:** DNA sequencing, Genetics research

## Abstract

Asymptomatic hyper-CK-emia (ASCK) is characterized by persistent elevation of creatine kinase (CK) in serum without any neurological symptoms. We ascertained a two-generation family of ASCK patients without clear neurological abnormalities except for the high levels of serum CK (810.5 ± 522.4 U/L). We identified a novel 1-bp deletion variant in the *DAG1* gene shared by the patients in the family (NM_001177639: exon 3: c.930delC:p.R311Gfs*70). The variant causes premature termination of translation at codon 477, resulting in a protein product completely devoid of the essential DAG1 domain. Since ASCK has been associated with *DAG1* in only one case carrying compound heterozygous missense variants, our new finding of a novel 1-bp deletion revealed the previously unknown dominant effect of *DAG1* on ASCK.

Serum creatine kinase (CK) levels are clinically important for the diagnosis of patients with muscle weakness, myopathy, or rhabdomyolysis. However, persistent and abnormally high levels of serum CK are occasionally observed in normal persons without any symptoms, which is termed aymptomatic hyper-CK-emia (ASCK). Although most ASCK cases, including idiopathic hyper-CK-emia (IHCK), are known to be sporadic, more than 20 pedigrees have been reported to be familial^1–11^.

We studied a two-generation Japanese pedigree of ASCK from Hiroshima Prefecture, a western province of Japan. The mode of inheritance in the ASCK pedigree is suggested to be autosomal dominant by the presence of male to male transmission (Fig. 1). All family members had no inconvenience in their daily activities. Three children (II-1, II-2, II-3) were unable to do a back-hip circle. All four affected members (I-1, II-1, II-2, II-3) had poor long-distance running. They had no clear neurological abnormalities except for the elevated levels of serum CK (810.5 ± 522.4 U/L). The ages of three children (II-1, II-2, II-3) were 10, 11, and 14 years old at the time of blood collection, respectively.

**Fig. 1:**
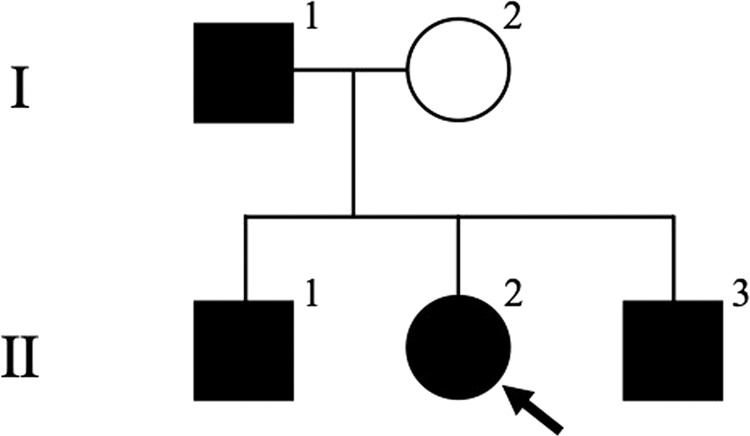
Pedigree of the tested family of asymptomatic hyper-CK-emia. Squares: males, circles: females, solid symbols: affected individuals, open symbols: unaffected individuals. The proband (II-2) is indicated with an arrow.

Whole-exome sequencing was carried out for two affected family members, II-2 and II-3, with sequencing depths of 69x and 50x, respectively (Fig. 1). Out of 124,432 single nucleotide variants (SNVs) identified in total, we selected 31,533 SNVs shared by both patients. We identified 315 SNVs located in 127 candidate genes of IHCK, malignant hyperthermia, and muscular dystrophy, including 151 functional SNVs (Supplementary Table 1). We excluded variants with frequencies larger than 0.002 in the 1000 G Project [http://www.1000genomes.org], Ensembl [http://asia.ensembl.org/index.html], HGVD [http://www.hgvd.genome.med.kyoto-u.ac.jp/], ToMMo [http://www.megabank.tohoku.ac.jp/], and ExAC [http://exac.broadinstitute.org/] databases, with the remaining five SNVs located in four genes (*DAG1*, *RYR1*, *SYNE1*, *TTN*). By Sanger sequencing using the primers shown in Supplementary Table 2, we identified only one SNV validated and cosegregated in the pedigree, which was a novel 1-bp deletion variant in exon 3 in the *DAG1* gene, NM_001177639:exon3:c.930delC:p.R311Gfs*70. The SNV is expected to cause a frameshift and premature termination of translation at codon 477 (Fig. 2, Supplementary Figure 1). We confirmed the absence of the SNV in 506 unrelated Japanese controls, indicating a frequency < 0.099% in the Japanese normal population.

**Fig. 2:**
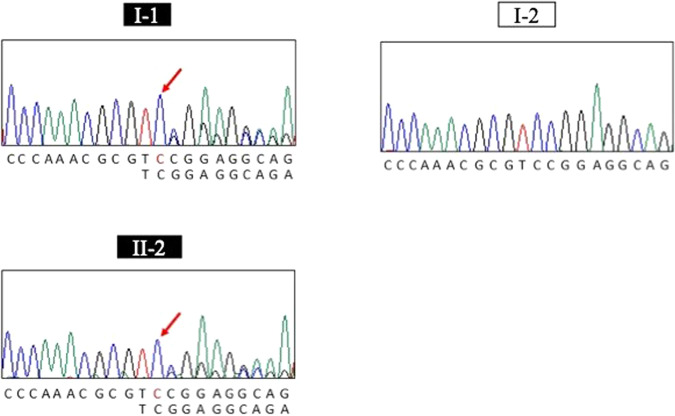
Electropherogram of the region of the variant NM_001177639: exon 3: c.930delC [p. R311Gfs*70] in two affected (I-1 and II-2) and one unaffected (I-2) family member. The locations of the variants are indicated by red arrows.

The *DAG1* gene encodes dystroglycan protein (NP_004384, Fig. 3), which is a dystrophin-associated glycoprotein (DAG) that is known to be responsible for muscular dystrophy (MD) and muscle-eye-brain disease^12,13^. Although the elevation of serum CK is commonly observed in MD patients, the current pedigree shows no neurological abnormalities related to MD. The premature termination of the translation caused by the 1-bp deletion in *DAG1* results in a protein product completely devoid of the DAG1 domain, which is known to be essential for *DAG1* function (Fig. 3)^14,15^. Since ASCK has been associated with *DAG1* in only one case carrying compound heterozygous missense variants in *DAG1*, our new finding of a novel 1-bp deletion shows the previously unknown dominant effect of *DAG1* for ASCK^16^. Dong et al. (2015) also observed very mild MD diagnosed only by immunohistochemistry^16^. The current patients, therefore, may show a similar subclinical MD, although immunohistochemical examination is not applicable due to the lack of muscle tissue samples. According to the ACMG/AMP/CAP guidelines, the 1-bp deletion variant in *DAG1* is classified as “pathogenic”, meeting the criteria of PVS1, PM1, PM2, PM4, PP1, and PP2. Therefore, we conclude that the novel 1-bp deletion in *DAG1*, NM_001177639:exon3:c.930delC:p. R311Gfs*70 is the causative dominant variant for the ASCK family.

**Fig. 3:**
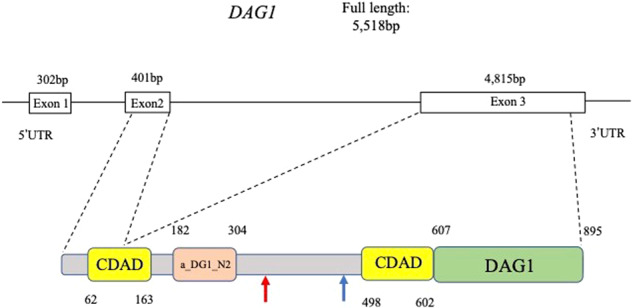
Genomic structure of the *DAG1* gene with domains and important functional regions of the DAG1 protein. Locations of the cadherin-like repeat domains of alpha dystroglycan (CADA), alpha-dystroglycan N-terminal domain 2 (a_DG1_N2), dystrophin-associated glycoprotein 1 (DAG1) are shown according to the Conserved Domains Database (CDD) (https://www.ncbi.nlm.nih.gov/Structure/cdd/cdd.shtml) and the Subfamily Protein Architecture Labeling Engine (SPARCLE) (https://www.ncbi.nlm.nih.gov/Structure/sparcle/docs/sparcle_about.html). Red and blue arrows indicate the locations of the variant and the premature stop codon, respectively.

## HGV Database

The relevant data from this Data Report are hosted at the Human Genome Variation Database at 10.6084/m9.figshare.hgv.3125.

## Supplementary information

Supplementary Table 1

Supplementary Table 2
