# Supplementary material for: A novel 1-bp deletion variant in DAG1 in Japanese familial asymptomatic hyper-CK-emia
Source: Hum Genome Var. 2022 Jan 27;9:4. doi: 10.1038/s41439-022-00182-0 (PMC8791931; doi:10.1038/s41439-022-00182-0)
Supplement: Supplementary file 1 — Supplementary Table 1 [file 41439_2022_182_MOESM1_ESM.pdf]

Supplementary Table1. Genes known to be associated with hyper CK-emia.

Gene	MIM number	Reference
ACADVL	609575	Aoyama T. et al., J. Clin. Invest. 95: 2465-2473, 1995.
ACTA1	102610	Laing N. G. et al., Human Mutation. 30(9):1267-1277, 2010.
AGL	610860	Aoyama Y. et al., J. Hum. Genet. 54: 681-686, 2009.
ALG13	300776	Dimassi S. et al., Clin. Genet. 89: 198-204, 2016.
ANO5	608662	Bolduc V. et al., Am. J. Hum. Genet. 86: 213-221, 2010.
ATP2A1	108730	Brody I. A. et al., New Eng. J. Med. 281: 187-192, 1969.
ATP7A	300011	Takata R. I. et al., J. Med. Genet. 41: 224-229, 2004.
B3GALNT2	610194	Stevens E. et al., Am. J. Hum. Genet. 92: 354-365, 2013.
B3GNT1	605517	Buysse K. et al., Hum. Molec. Genet. 22: 1746-1754, 2013.
BAG3	603883	Selcen D. et al., Ann. Neurol. 65: 83-89, 2009.
BIN1	601248	Nicot A.-S. et al., (Letter) Nature Genet. 39: 1134-1139, 2007.
CACNA1S	114208	Boerman R. H. et al., J. Med. Genet. 32: 44-47, 1995.
CAPN3	114240	Richard I. et al., Cell 81: 27-40, 1995.
CAV3	601253	Fulizio L. et al., Hum. Mutat. 25: 82-89, 2005.
CFL2	601443	Agrawal P. B. et al., Am. J. Hum. Genet. 80: 162-167, 2007.
CHKB	612395	Sher R. B. et al., J. Biol. Chem. 281: 4938-4948, 2006.
ClC1	118425	Koch M. C. et al., Science 257: 797-800, 1992.
CNTN1	600016	Compton A. G. et al., Am. J. Hum. Genet. 83: 714-724, 2008.
COL6A1	120220	Jobsis G. J. et al., Nature Genet. 14: 113-115, 1996.
COL6A2	120240	Jobsis G. J. et al., Nature Genet. 14: 113-115, 1996.
COL6A3	120250	Baker N. L. et al., Hum. Molec. Genet. 14: 279-293, 2005.
COQ10	607426	Quinzii C. et al., Developmental Disabilities Research Reviews. 16(2):183, 2010.
CPT1B	601987	Britton C. H. et al., Genomics 40: 209-211, 1997.
CPT2	600650	Bonnefont J.-P. et al., Am. J. Hum. Genet. 58: 971-978, 1996.
CRPPA	614631	Willer T. et al., Nature Genet. 44: 575-580, 2012.
CRYAB	123590	Fardeau M. et al., Rev. Neurol. 134: 411-425, 1978.
DAG1	128239	Gee S. H. et al., Cell 77: 675-686, 1994.
DES	125660	Ariza A. et al., Hum. Path. 26: 1032-1037, 1995.
DMD	300377	Abbs S. et al., Genomics 7: 602-606, 1990.
DMPK	605377	Fu Y.-H. et al., Science 255: 1256-1258, 1992.
DNM2	602378	Bitoun M. et al., Nature Genet. 37: 1207-1209, 2005.
DOLK	610746	Lefeber D. J. et al., PLoS Genet. 7: e1002427, 2011.
DPM1	603503	Yang A. C. et al., Molec. Genet. Metab. 110: 345-351, 2013.
DPM2	603564	Barone R. et al., Ann. Neurol. 72: 550-558, 2012.
DPM3	605951	Lefeber D. J. et al., Am. J. Hum. Genet. 85: 76-86, 2009.
DUX4	606009	Lemmers RJ et al., Science. 329(5999):1650-1653, 2010.
DYSF	603009	Weiler T. et al., Am. J. Hum. Genet. 59: 872-878, 1996.
EMD	300384	Bione S. et al., Hum. Molec. Genet. 4: 1859-1863, 1995.
ENO3	131370	Comi G. P. et al., Ann. Neurol. 50: 202-207, 2001.
ETF	615256	Cloutier P. et al., PLoS Genet. 9: e1003210, 2013.
FHL1	300163	Windpassinger C. et al., Am. J. Hum. Genet. 82: 88-99, 2008.
FKRP	606596	Mercuri E. et al., Neuropediatrics 31: 186-189, 2000.
FKTN/ FCMD	607440	Colombo R. et al., Hum. Genet. 107: 559-567, 2000.
FLNC	102565	Thompson T. G. et al., J. Cell Biol. 148: 115-126, 2000.
GAA	606800	Wokke J. H. J. et al., Ann. Neurol. 38: 450-454, 1995.
GBE1	607839	Tang T. T. et al., Acta Neuropath. 87: 531-536, 1994.
GMPPB	615320	Carss K. J. et al., Am. J. Hum. Genet. 93: 29-41, 2013.
GNE	603824	Nonaka I. et al., J. Neurol. Sci. 51: 141-155, 1981.
GTDC2	614828	Manzini M. C. et al., Am. J. Hum. Genet. 91: 541-547, 2012.
GYG1	603942	Malfatti E. et al., Ann. Neurol. 76: 891-898, 2014.
GYS1	138570	Kollberg G. et al., New Eng. J. Med. 357: 1507-1514, 2007.
HNRNPDL	607137	Vieira N. M. et al., Hum. Molec. Genet. 23: 4103-4110, 2014.
HNRPDL	607137	Nigro V. et al., Acta Myologica. 33(1):1-12, 2014.
ISPD	614631	Willer T. et al., Nature Genet. 44: 575-580, 2012.
ITGA7	600536	Hayashi Y. K. et al., Nature Genet. 19: 94-97, 1998.
KBTBD13	613727	Sambuughin N. et al., Am. J. Hum. Genet. 88: 122, 2011.
KLHL40	615340	Ravenscroft G. et al., American Journal of Human Genetics. 93(1):6-18, 2013.
LAMA2	156225	Helbling-Leclerc A. et al., Nature Genet. 11: 216-218, 1995.
LAMP2	309060	Charron P. et al., Heart 90: 842-846, 2004.
LARGE1	603590	Barresi R. et al., Nature Med. 10: 696-703, 2004.
LDB3	605906	Selcen D. et al., Ann. Neurol. 57: 269-276, 2005.
LDHA	150000	Maekawa M. et al., Am. J. Hum. Genet. 39: 232-238, 1986.
LIMS2	607908	Warman Chardon et al., Clin. Genet. 88: 558-564, 2015.
LMNA	150330	Haque F. et al., J. Biol. Chem. 285: 3487-3498, 2010.
LMOD3	616112	Yuen M. et al., The Journal of Clinical Investigation. 124(11):4693-4708, 2014.
MEGF10	612453	Logan C. V. et al., Nature Genet. 43: 1189-1192, 2011.
MT-CYB	516020	Massie R. et al., Muscle & Nerve. 42(1):136-140, 2010.
MTM1	300415	Buj-Bello A. et al., Hum. Mutat. 14: 320-325, 1999.
MYBPC3	600958	Watkins H. et al., Nature Genet. 11: 434-437, 1995.
MYH2	160740	Martinsson T. et al., Proc. Nat. Acad. Sci. 97: 14614-14619, 2000.
MYH7	160760	Tajsharghi H. et al., Ann. Neurol. 54: 494-500, 2003.
MYL1	160780	Ravenscroft G. et al., Hum. Molec. Genet. 27: 4263-4272, 2018.
MYO18B	607295	Nishioka M. et al., Proc. Nat. Acad. Sci. 99: 12269-12274,2002.
MYOT	604103	Hauser M. A. et al., Hum. Molec. Genet. 9: 2141-2147, 2000.
MYPN	608517	Miyatake S. et al., American Journal of Human Genetics. 100(1):169-178, 2017.
NEB	161650	Pelin K. et al., Proc. Nat. Acad. Sci. 96: 2305-2310, 1999.
OCTN2/ SLC22A5	603377	Liang W. et al., Current Neurology and Neuroscience Reports. 11(1):97-103, 2011.
PDE9A	602973	Lee, D. I. et al., Nature 519: 472-476, 2015.
PFKM	610681	Hamaguchi T. et al., Hum. Mutat. 8: 273-275, 1996.
PGAM2	612931	Edwards Y. H. et al., Genomics 5: 948-951, 1989.
PGK1	311800	Fujii H. et al., J. Biol. Chem. 255: 6421-6423, 1980.
PGM1	171900	Stojkovic T. et al., (Letter) New Eng. J. Med. 361: 425-427, 2009.
PHKA1	311870	Clemens P. R. et al., Ann. Neurol. 28: 529-538, 1990.
PLEC1	601282	Gundesli H. et al., Am. J. Hum. Genet. 87: 834-841, 2010.
PNPLA2	609059	Fischer J. et al., Nature Genetics. 39(1):28-30, 2007.
POGLUT1	615618	Servian-Morilla E. et al., EMBO Molec. Med. 8: 1289-1309, 2016.
POLG1	174763	Milone M. et al., The Neurologist. 16(2):84, 2010.
POMGNT1	606822	Yoshida A. et al., Dev. Cell 1: 717-724, 2001.
POMGNT2	614828	Yoshida-Moriguchi T. et al., Science 341: 896-899, 2013.
POMT1	607423	Beltran-Valero de Bernabe D. et al., Am. J. Hum. Genet. 71: 1033-1043, 2002.
POMT2	607439	van Reeuwijk J. et al., J. Med. Genet. 42: 907-912, 2005.
POPDC1/ BVES	604577	Bonne et al., Neuromuscular Disorders. 27, 2017.
PPA2	609988	Kennedy H. et al., Am. J. Hum. Genet. 99: 674-682, 2016.
PTRF	603198	Hayashi Y. K. et al., J. Clin. Invest. 119: 2623-2633, 2009.
PYGM	608455	Kubisch C. et al., Hum. Mutat. 12: 27-32, 1998.
PYROXD1	617220	O'Grady G. L. et al., Am. J. Hum. Genet. 99: 1086-1105, 2016.
RRM2B	604712	Tyynismaa H. et al., Am. J. Hum. Genet. 85: 290-295, 2009.
RXYLT1	605862	Vuillaumier-Barrot S. et al., Am. J. Hum. Genet. 91: 1135-1143, 2012.
RYR1	180901	Brandt A. et al., Hum. Molec. Genet. 8: 2055-2062, 1999.
RYR3	180903	Sorrentino V. et al., Genomics 18: 163-165, 1993.
SCN4A	603967	Palma C. et al., Neuromuscular Disorders. 27(12):1123-1125, 2017.
SEPN1	606210	Ferreiro A.et al., Am. J. Hum. Genet. 71: 739-749, 2002.
SGCA	600119	Romero N. B. et al., C. R. Acad. Sci. III 317: 70-76, 1994.
SGCB	600900	Lim L. E. et al., Nature Genet. 11: 257-265, 1995.
SGCD	601411	Nigro V. et al., Nature Genet. 14: 195-198, 1996.
SGCG	608896	Ben Othmane K. et al., Nature Genet. 2: 315-317, 1992.
SLC25A20	613698	Huizing M. et al., Am. J. Hum. Genet. 61: 1239-1245, 1997.
SMCHD1	614982	Lemmers R. J. L. F. et al., Nature Genet. 44: 1370-1374, 2012.
SUCLA2	603921	Elpeleg O. et al., Am. J. Hum. Genet. 76: 1081-1086, 2005.
SUN1	607723	Chen C.-Y. et al., Cell 149: 565-577, 2012.
SUN2	613569	Haque F. et al., J. Biol. Chem. 285: 3487-3498, 2010.
SYNE1	608441	Zhang Q. et al.,Hum. Molec. Genet. 16: 2816-2833, 2007.
SYNE2	608442	Zhang Q. et al.,Hum. Molec. Genet. 16: 2816-2833, 2007.
TCAP	604488	Moreira E. S. et al., Nature Genet. 24: 163-166, 2000.
TK2	188250	Roos S. et al., Neuromuscular Disorders. 24(8):713-720, 2014.
TMEM43	612048	Liang W. C. et al., Ann. Neurol. 69: 1005-1013, 2011.
TNNT1	191041	Johnston J. J. et al., Am. J. Hum. Genet. 67: 814-821, 2000.
TNNT3	600692	Zhao N. et al., Europ. J. Med. Genet. 54: 351-353, 2011.
TNPO3	610032	Melia M. J. et al., Brain 136: 1508-1517, 2013.
TOR1AIP1	614512	Kayman-Kurekci G. et al.,Neuromusc. Disord. 24: 624-633, 2014.
TPM2	190990	Donner K. et al., Neuromusc. Disord. 12: 151-158, 2002.
TPM3	191030	Laing N. G. et al., Nature Genet. 10: 249, 1995.
TRAPPC11	614138	Bogershausen N. et al., Am. J. Hum. Genet. 93: 181-190, 2013.
TRDN	603283	Oddoux S. et al., J. Biol. Chem. 284: 34918-34929, 2009.
TRIM32	602290	Frosk P. et al., Am. J. Hum. Genet. 70: 663-672, 2002.
TTN	188840	Hackman P. et al., Am. J. Hum. Genet. 71: 492-500, 2002.
VCP	601023	Watts G. D. J. et al., Nature Genet. 36: 377-381, 2004.
