# Supplementary material for: A novel 1-bp deletion variant in DAG1 in Japanese familial asymptomatic hyper-CK-emia
Source: Hum Genome Var. 2022 Jan 27;9:4. doi: 10.1038/s41439-022-00182-0 (PMC8791931; doi:10.1038/s41439-022-00182-0)
Supplement: Supplementary file 2 — Supplementary Table 2 [file 41439_2022_182_MOESM2_ESM.pdf]

Supplementary Table2. Primers used in Sanger validation.

Gene	GeneBank Accession Number	Sequence (5' to 3')	Genomic location (exon)	Product size (bp)
DAG1	NM_001177639.2	AAATTAGTGCCGGTGGTGAA	3	475
		CCTAGGGTTGGGGTTTGAAT		
RYR1	NM_000540.3	CAGCCTCAGTTTCCCAAAGT	90	850
		GCCTCCTCCTCGTCTCCAG		
SYNE1	NM_182961.4	TTGACTACACCCCTCTCTGGA	27	348
		TTGGTAGTCTGCGTGGGAAT		
		AGGTGGATCAATTCTTACCTTTC	35	477
		CCGTATGCTCTATCCAGCAAG		
TTN	NM_133378.4	CCAACTCCAGCAGCATTTTC	275	478
		CTTCAAGATGGTGGCAGTGA		
